# No Evidence of Ntwetwe Virus Infections in Children Presenting to Kiboga Hospital, Uganda

**DOI:** 10.3390/tropicalmed8010021

**Published:** 2022-12-27

**Authors:** Arthur W. D. Edridge, Nathalie van den Brekel, Philly Mukungu, Rachael Nakayima, Samuel Bbosa, Peter Isagara, Michael Boele van Hensbroek, Lia van der Hoek, John Kayiwa, Julius J. Lutwama, Richard Idro

**Affiliations:** 1 Center for Global Child Health, Emma’s Children Hospital, Amsterdam UMC, Location AMC, University of Amsterdam, 1105 AZ Amsterdam, The Netherlands; 2 Virus Discovery Group, Laboratory of Experimental Virology, Department of Medical Microbiology and Infection Prevention, Amsterdam UMC, Location AMC, University of Amsterdam, 1105 AZ Amsterdam, The Netherlands; 3 Kiboga District Hospital, Kiboga P.O. Box 17, Uganda; 4 Department of Arbovirology, Ugandan Virus Research Institute, Entebbe P.O. Box 49, Uganda; 5 Department of Paediatrics, Mulago Hospital, College of Health Science, Makerere University, Kampala P.O. Box 7051, Uganda

**Keywords:** orthobunyavirus, CNS infection, virus discovery, emerging infectious diseases, Uganda, pediatrics

## Abstract

We investigated whether Ntwetwe virus—a novel orthobunyavirus discovered in a Ugandan girl with a fatal encephalopathy—was a common reason for hospital admission for children to Kiboga hospital, Uganda. A case–control was conducted between September 2019 and September 2020, including cases with severe neurological disease and mild febrile illness, matched to a healthy control without fever. Among 143 subjects, no cases with an acute infection were identified. This result suggests that Ntwetwe virus does not cause a major burden of disease amongst children presenting to Kiboga hospital during the study period.

## 1. Introduction

Orthobunyaviruses are a group of arthropod-borne RNA viruses that can cause a wide range of disease in humans, including severe central nervous system infections [1]. Recently, we discovered a novel orthobunyavirus named Ntwetwe virus in the cerebrospinal fluid of a Ugandan girl with fatal encephalopathy [2]. This discovery suggests that Ntwetwe virus may have been the cause of disease, yet further studies to evaluate the proof of causality are warranted [3]. If so, human exposure to this virus may be common, as the virus is probably transmitted by *Anopheles* mosquitoes, which are highly prevalent in Uganda and many other countries [2]. We therefore aimed to further evaluate the proof of causality and determine the potential burden of disease caused by Ntwetwe virus in Kiboga, Uganda, the area where the index case came from [2].

## 2. Materials and Methods

We conducted a case–control study enrolling children with mild arboviral-like and severe neurologic disease from Kiboga hospital, Kiboga, Uganda (in- and exclusion criteria in Table 1). All cases were age-matched to a healthy control (a siblingp of a case or another patient presenting to the hospital in the same week). Medical history and data on epidemiological risk factors for infection were recorded, and blood and cerebrospinal fluid (severe cases only) samples were collected. The study was conducted for one full year (September 2019–September 2020) to allow for potential seasonal variation in disease transmission. All children presenting with severe neurologic disease were considered for inclusion. Because presentation with mild arboviral-like disease was common, we included up to three mild cases per week, limiting the number of inclusions while still evaluating season variability. 

The study was approved by the Ugandan National Council for Science and Technology (HS 2603) and Makerere University School of Medicine Research and Ethic Committee (REC REF 2019-075). Informed consent was obtained from the guardians of all cases and controls. 

In addition to the Kiboga study, samples from 29 children (including the index case, included between November 2015 and June 2016, age range 6 months to 12 years) with non-traumatic coma and/or convulsive status epilepticus from Mulago national referral hospital, Kampala, from a previous study were also included for analysis [2].

To evaluate acute Ntwetwe virus infections, the presence of Ntwetwe virus RNA was evaluated in plasma and cerebrospinal fluid using a reverse transcription quantitative polymerase chain reaction (RT-qPCR, limit of detection of 250 RNA copies per reaction) [2]. 

## 3. Results

Between September 2019 and September 2020, 9 severe neurologic cases, 95 mild cases and 97 controls (201 total) were enrolled from Kiboga hospital. Patient characteristics are described in Table 2. Sufficient sample volumes from all severe, 71 mild cases, and 63 controls (143 total) children from Kiboga hospital, and 29 from Mulago hospital Kampala, were available for analysis. Analysis by RT-qPCR to detect viral RNA revealed no positive samples beside the index case (positive control). 

## 4. Discussion

We aimed to determine the potential burden of disease caused by Ntwetwe virus in the area where the index case came from. Our findings indicate that Ntwetwe virus may not be causing a major burden of severe disease among children in Kiboga district, Uganda. We were unable to find any qPCR confirmed cases among cases with severe neurological (comparable to the index case) or mild arboviral-like disease. Moreover, during the period of one year, we did not observe an outbreak of severe neurologic cases, and hospital presentations were even lower than previously documented—about one per fortnight—resulting in only nine inclusions of severe neurologic disease. It should be noted that the emergence of COVID-19 during the study inclusion period severely decreased hospital admission in this area, probably because of decreased human-to-human transmission of infectious diseases and higher barrier to healthcare-seeking behaviour in general [4]. However, this likely did not affect hospital presentation with Ntwetwe virus infections, as humans generally are dead-end hosts for orthobunyaviruses [5], and severe neurological cases will probably still present to hospital because of the severity of the disease. 

We attempted to also study prior infections using a partial viral glycoprotein ELISA assay, yet we could not validate the results as the index case remained seronegative for both IgM and IgG. As the coding sequence of the glycoprotein has only partially been uncovered, further genomic characterisation is required before a potentially more sensitive assay can be constructed.

Future studies may primarily focus on obtaining a new isolate of the virus. This would not only allow complete genomic characterization, but also aid in further evaluating the causality of neurologic disease, e.g., through experimental animal or brain organoid infection studies [3]. Currently, we do not advocate the implementation of larger prospective studies primarily focused on evaluating the burden of Ntwetwe virus, yet we recommended that research efforts that are already focusing on the aetiology of neurological and/or arboviral diseases in Uganda include Ntwetwe virus in their diagnostic testing panels, either through targeted qPCR or untargeted metagenomic studies. 

## Figures and Tables

**Table 1:** In- and exclusion criteria.

Criteria	Severe Neurologic Case	Mild Arboviral-like Case	Healthy Control
Age	>6 months and <18 years	>6 months and <18 years	>6 months, <18 years and +/−2.5 years of matched case’s age
Axillary temperature	≥37.5 °C	≥37.5 °C	<37.5 °C
Symptoms	One of: -Blantyre coms scale <3 for 30 min -Convulsions >5 min -Convulsion unresponsive to two doses of first-line anticonvulsant -Meningeal signs	One of: -Severe headache -Severe muscle pain -Severe abdominal pain -Non-traumatic bleeding -Photophobia -Erythematous maculopapular rash -Confusion -Hallucinations -Convulsions -Blantyre coma scale <5 for 30 min	None
Exclusion criteria	Any of: -Restoration of consciousness upon correction of hypoglycaemia -Pre-existing neurologic condition		Any symptom required for severe neurologic or mild case definition
Inclusion frequency	All	Up to three per week	When a case is enrolled

**Table 2:** Patient characteristics.

	Kiboga Severe	Kiboga Mild	Kiboga Control	Kampala Severe ^$^
Total inclusions	9	95	97	29
Age, median (1st–3rd quantile)	3.8 (2–5)	4 (2–6)	4.1 (2–8)	3 (2–5)
Male sex, N (%)	5 (56%)	35 (37%)	41 (42%)	19 (66%)
Symptoms and signs, N (%)				
-Severe headache	6 (67%)	75 (79%)	15 (15%)	NA
-Confusion	4 (44%)	1 (1%)	0 (0%)	NA
-Seizures	9 (100%)	26 (27%)	0 (0%)	24 (83%)
-Seizure lasting for >5 min	2 (22%)	0 (0%)	0 (0%)	10 (34%)
-Seizure not responding to two doses anticonvulsants	6 (67%)	0 (0%)	0 (0%)	12 (41%)
-Severe muscle pain	0 (0%)	2 (2%)	0 (0%)	NA
-Severe Abdominal pain	6 (67%)	82 (86%)	6 (6%)	NA
-Bleeding (not related to injury)	0 (0%)	1 (1%)	0 (0%)	NA
-Blantyre coma scale <3	2 (22%)	0 (0%)	0 (0%)	27 (93%)
-Meningeal signs	1 (11%)	0 (0%)	0 (0%)	4 (14%)

^$^ Children presenting to Mulago hospital in Kampala. Children did not necessarily become ill in Kampala as it is the national referral center.

## Data Availability

Not applicable.
